# Exploring eukaryotic formate metabolisms to enhance microbial growth and lipid accumulation

**DOI:** 10.1186/s13068-017-0708-1

**Published:** 2017-01-26

**Authors:** Zhiguo Liu, Tolutola Oyetunde, Whitney D. Hollinshead, Anna Hermanns, Yinjie J. Tang, Wei Liao, Yan Liu

**Affiliations:** 1 Department of Biosystems and Agricultural Engineering, Michigan State University, East Lansing, MI 48824-1323 USA; 2 Department of Energy, Environmental and Chemical Engineering, Washington University in St. Louis, St. Louis, MO 63110 USA; 3 Department of Biology, Heinrich Heine University, 40225 Düsseldorf, Germany

**Keywords:** ^13^C, NAD(P)H, Fatty acids, Flux balance analysis, One-carbon metabolic pathway

## Abstract

**Background:**

C1 substrates (such as formate and methanol) are promising feedstock for biochemical/biofuel production. Numerous studies have been focusing on engineering heterologous pathways to incorporate C1 substrates into biomass, while the engineered microbial hosts often demonstrate inferior fermentation performance due to substrate toxicity, metabolic burdens from engineered pathways, and poor enzyme activities. Alternatively, exploring native C1 pathways in non-model microbes could be a better solution to address these challenges.

**Results:**

An oleaginous fungus, *Umbelopsis isabellina*, demonstrates an excellent capability of metabolizing formate to promote growth and lipid accumulation. By co-feeding formate with glucose at a mole ratio of 3.9:1, biomass and lipid productivities of the culture in 7.5 L bioreactors were improved by 20 and 70%, respectively. ^13^C-metabolite analysis, genome annotations, and enzyme assay further discovered that formate not only provides an auxiliary energy source [promoting NAD(P)H and ATP] for cell anabolism, but also contributes carbon backbones via folate-mediated C1 pathways. More interestingly, formate addition can tune fatty acid profile and increase the portion of medium-chain fatty acids, which would benefit conversion of fungal lipids for high-quality biofuel production. Flux balance analysis further indicates that formate co-utilization can power microbial metabolism to improve biosynthesis, particularly on glucose-limited cultures.

**Conclusion:**

This study demonstrates *Umbelopsis isabellina’s* strong capability for co-utilizing formate to produce biomass and enhance fatty acid production. It is a promising non-model platform that can be potentially integrated with photochemical/electrochemical processes to efficiently convert carbon dioxide into biofuels and value-added chemicals.

## Background

Microbial one-carbon (C1) metabolism plays a critical role in global carbon cycles [1]. Key C1 molecules include carbon dioxide, methane, carbon monoxide, and methanol. Among these C1 compounds, CO_2_ is one of the most abundant greenhouse gases in the Earth’s atmosphere, which contributes up to 26% of the global greenhouse effect [2]. Developing technologies to fix and utilize CO_2_ has attracted increasing attention to alleviate the impact of greenhouse gas emissions on climate change. Numerous studies have been conducted on microbial C1 metabolism, such as autotrophic CO_2_ fixation, and methylotrophic or methanotrophic carbon utilization (from CO_2_ reduction reaction) [3–5]. In general, autotrophic CO_2_ fixation faces challenges including mass transfer limitation, low metabolic rate of adenosine triphosphate (ATP)/reduced nicotinamide adenine dinucleotide [NAD(P)H] generations, and poor enzymatic synthesis activities. Alternatively, formate, a stable C1 compound [6], can be generated from CO_2_ using electrochemical reduction under mild reaction conditions and proper catalysts [6, 7]. Due to its solubility and reducing power, formate is a promising feedstock to support microbial growth.

Bacterial formate utilization has been intensively studied in methylotrophs and lithoautotrophs [7–10]. There are two interdependent processes naturally existed to achieve formate metabolism: formate oxidation and formate assimilation [11–13]. The formate oxidation relies on NAD(P)H-dependent formate dehydrogenase (FDH) that transfers electrons from formate to NAD(P)H, and facilitates ATP synthesis to support cell growth [14–16]. For example, *Ralstonia eutropha* uses the formate oxidation to gain ATP and NAD(P)H, and to support the Calvin Cycle to fix CO_2_ [1]. In contrast, the formate assimilation directly incorporates formate via five possible pathways including the serine pathway, the reductive acetyl-CoA pathway, the ribulose monophosphate pathway, the xylulose 5-phosphate pathway, and the reductive glycine pathway [1, 17]. All of these pathways start from the reduction of formate to methylenetetrahydrofolate (methylene-THF). For example, some methylotrophs first convert formate into formyl-THF that is reduced to methylene-THF. The methylene-THF then donates its formaldehyde for serine generation, and leads to production of acetyl-CoA [1]. According to these theoretical analyses, metabolic engineers have been devoting tremendous efforts to develop model bacterial platform for C1 utilization [18–20]. *Corynebacterium glutamicum* and *E. coli* were two of the model strains that have been engineered to co-utilize C1 substrates, but bacterial platform shows deleterious growth and poor carbon utilizations.

As for eukaryotes, a few species can tolerate and utilize formate, and most of them use formate oxidation pathway to release NAD(P)H from formate to support their growth [21–25]. For instance, with the addition of formate, Penicillin G production of *Penicillium chrysogenum* fermentation was significantly improved [26]. In this study, we focus on an oleaginous fungus, *Umbelopsis isabellina*, that has demonstrated the capability to co-utilize formate and other carbon sources for growth and lipid accumulation. To explore fungal formate assimilation pathways and co-metabolism on diverse carbon substrates, this study has (1) used ^13^C tracing to elucidate formate utilization pathways in *U. isabellina*, (2) applied biochemistry assay to delineate the effects of formate on fungal growth and fatty acid accumulation, and (3) rigorously quantified C1 carbon contributions to biomass synthesis.

## Methods

### Strain, seed culture, and reagents

*Umbelopsis Isabellina* ATCC 42613 was obtained from the American Type Culture Collection (Manassas, VA). Spores were washed by sterile deionized water and collected after cultivation on potato dextrose agar (PDA) medium at 30 °C for two weeks. Seed was cultured in 24 g L^−1^ potato dextrose broth (PDB) medium with 8 g L^−1^ yeast extract (YE) (Neogen, Lansing), at room temperature of 25 °C and a shaking speed of 180 rpm for 36 h. Inoculation size was 5% (v/v) if not specified. Chemicals, substrates (except ^13^C-formate from Cambridge Isotope Laboratories, Inc., Tewksbury, MA), and reagents were purchased from Sigma-Aldrich (Sigma-Aldrich, St. Louis, MO).

### Fermentation

Batch fermentation was conducted using shaking flasks and fermenters. For the shaking flask culture, 2-L Erlenmeyer flasks containing 0.7 L medium were placed on a New Brunswick Shaker at 180 rpm and cultured at 25 °C. For the fermenter culture, 7.5-L fermenters (New Brunswick–Eppendorf, Bioflo 115, Hauppauge, NY) were used to carry out the experiment. The fermenters contained 3 L medium. The airflow rate was 1 vvm, and the agitation speed was 200 rpm. The flask culture of formate medium contained 0.5 g L^−1^ YE, 2 g L^−1^ ^13^C-labeled formate, and trace elements [27]. The flask culture of glucose and formate medium contained 0.5 g L^−1^ YE, 4 g L^−1^ ^13^C-labeled formate (99% purity), 10 g L^−1^ glucose, and trace elements. The duration of all flask cultures was 72 h. The medium for the fermenter culture contained 1 g L^−1^ YE, 10 g L^−1^ glucose and 4 g L^−1^ ^13^C-labeled formate (the control experiment excluded formate supplement). The duration of fermenter culture was 48 h. The pH of the flask cultivation was maintained at 6.0 by manually adding 1 M sulfuric acid or sodium hydroxide every 6 h in first 12 h and every 12 h afterwards. The pH of the fermenter cultivation was automatically maintained at 6.0 using 1 M sulfuric acid or sodium hydroxide. Samples were taken periodically for biomass composition and metabolite measurement.

### Metabolite and biomass composition analyses

Formate and glucose in the fermentation broth were determined by a High-Performance Liquid Chromatography (HPLC) (Shimadzu Prominence, Japan) equipped with a Bio-rad Aminex HPX-87H analytical column and a refractive index detector [27]. As for NADH measurement, one ml broth was centrifuged at 4000 rpm for 1 min and the precipitated biomass was washed by cold phosphate buffered saline (PBS) before the NADH was measured by the NAD+/NADH Quantification Colorimetric Kit (BioVision, San Francisco). Standard solutions were prepared and measured on daily basis.

Fungal biomass with the known volume of fermentation broth was collected by vacuum filtration and washed three times by deionized water before drying at 105 °C overnight for dry matter measurement. Lipid extraction of the fungal biomass was conducted using the Bligh and Dyer method [28], and the lipid content was determined gravimetrically. The extracted lipid was then subjected to methanolysis to turn fatty acids into methyl esters [29]. The resulting methyl esters were further analyzed by gas chromatography–mass spectrometry (GC–MS) to obtain the fatty acid profile [27]. F.A.M.E. Mix (C4–C24) with serial dilutions (100, 50, 25, 12.5, 6.25, 3.125, 1.5625, 0.78125 μg mL^−1^) and chloroform was applied as external standard. Samples were diluted ten times prior to analysis to reach reasonable detection levels. All samples and standards were spiked with 50 μg mL^−1^ methyl nonadecanoate (C19:0) as internal standard.

### Carbon isotopomer analysis

Biomass samples were harvested by centrifuge, and the cell pellets were hydrolyzed with 6 mol L^−1^ HCl at 100 °C overnight. The resulting amino acid mixtures were subsequently air dried and derivatized with *N*-*tert*-butyldimethylsilyl-*N*-methyltrifluoroacetamide (TBDMS) prior to GC–MS analysis. A published software was used to analyze and correct amino acid MS data, as described in the previous report [30]. The fragments [M-57] or [M-15], containing the entire carbon backbone of the amino acids, were used to demonstrate the incorporation of ^13^C-formate. Results are described using *m* + 0, *m* + 1, and *m* + 2 for unlabeled, singly labeled, doubly labeled amino acids, and so on.

### Flux balance analysis (FBA) of fungal formate co-utilization

To understand the influence of fungal formate metabolism on biomass growth, a genome-scale yeast model, iMM904 (bigg.ucsd.edu), was adapted to provide stoichiometric insights into formate–glucose co-metabolism [31]. The model was modified by adding a pathway from formate to methylene-THF. The growth rates were predicted based on different glucose/formate co-utilization influxes and the objective function of maximal biomass production. The FBA simulation using COBRA tool box can then determine the carbon flows from formate to biomass synthesis (formate assimilation) and to CO_2_ release for NAD(P)H generations (formate oxidation).

## Results and discussion

### Formate as an energy and carbon source to enhance the growth of *U. isabellina*

**Fig. 1:**
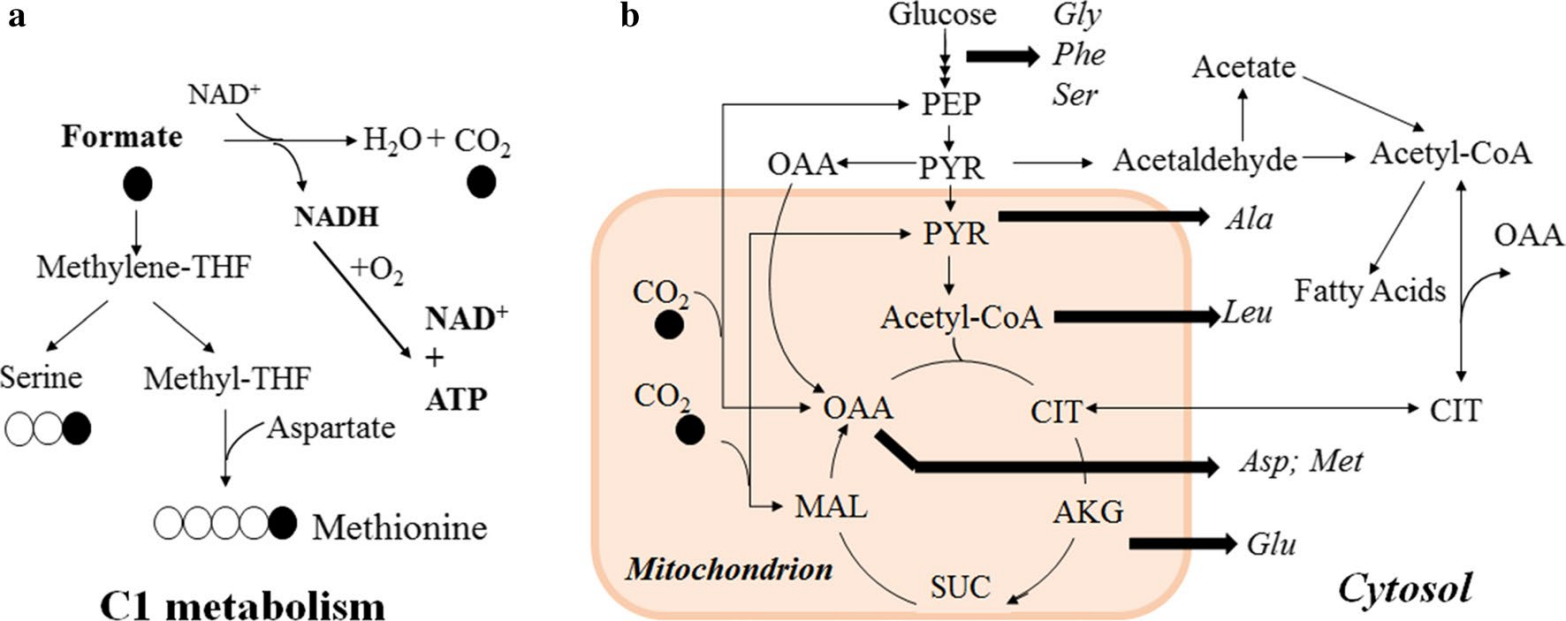
Simplified pathways of carbon and energy metabolism during *U. isabellina* culture on glucose and formate. **a** Formate metabolism. **b** Glucose metabolism

**Fig. 2:**
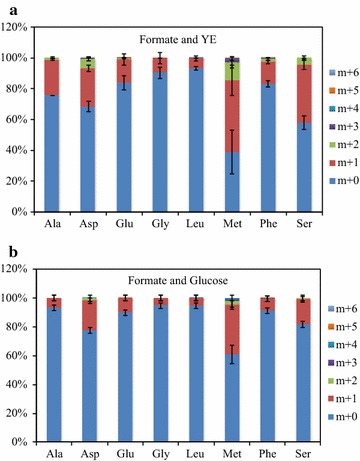
Contribution of formate carbon to amino acids in proteinogenic amino acids of *Umbelopsis Isabellina* with different carbon sources. Only amino acids with significant labeling pattern are displayed. Data are average of two replicates with standard error. **a** ^13^C-Formate as sole carbon source (yeast extract (YE) present in medium); **b** ^13^C-Formate + Glucose

The isotopomer analysis [32] provided the labeling information of critical precursor metabolites during formate assimilation such as pyruvate, acetyl-CoA, methylene-THF, formyl-THF, glycine, and serine (Fig. 1). The fungal culture on ^13^C-formate supplemented with yeast extract shows that eight amino acids (alanine, aspartate, glutamate, glycine, leucine, methionine, phenylalanine, and serine) were detected with significant labeling extent (Fig. 2a). Among them, methionine and serine are the amino acids with a large amount of *m* + 2 (e.g., over 10% of methionine was labeled with two carbons). Besides, methionine labeling enrichment is significantly (P < 0.05) higher than its precursor aspartate, indicating the labeling of Methyl-THF unit (Asp + Methyl-THF → methionine). This result demonstrates that *U. isabellina* assimilates ^13^C-formate via folate-mediated C1 pathways to generate Methyl-THF (Fig. 1). On the other hand, the TCA cycle-derived amino acids (aspartate and glutamate) show enriched ^13^C (15~30%, mostly *m* + 1), which indicates that the strain also uses anaplerotic pathway (PEP + CO_2_ → Oxaloacetate) to fix the CO_2_ from formate oxidation (Fig. 1). Similar labeling distributions were observed when adding formate in glucose medium (Fig. 2b). The ^13^C-fingerprinting provided the evidence of formate contribution to fungal growth through both formate assimilation and oxidation pathways. However, the experimental data also demonstrate that formate is not an efficient carbon source to support high-yield biomass growth (Table 1). The biomass accumulation on the formate medium was much lower than the culture on the glucose medium. Mixing formate with other carbon sources could be a good solution to synergistically utilize carbon and energy contents in formate and enhance carbon utilization efficiency of the fermentation. Therefore, the effects of formate as a supplemental carbon source on fungal metabolism was further studied.

**Table 1:** *U. isabellina* growth on the media with formate and glucose as individual carbon sources

	Biomass concentration (g L^−1^)		
Culture time (h)	0	48	96
Formate medium	0.31 ± 0.03	0.58 ± 0.04	0.58 ± 0.08
Glucose medium	0.21 ± 0.02	3.67 ± 0.43	7.58 ± 0.28

Data are average of three replicates with standard deviation

**Fig. 3:**
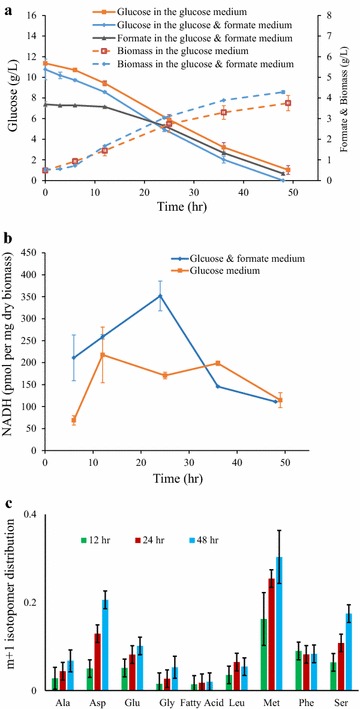
Kinetics of fermentor culture using ^13^C-formate and glucose as the carbon sources. Data are average of two replicates with standard error. **a** Growth and substrate consumption kinetics; **b** NADH level kinetics; **c** Mass isotopomer *m* + 1 fraction of proteinogenic amino acids for biomass collected at 12, 24, and 48 h. Fatty acid labeling estimated based on labeling of acetyl-CoA, which was determined from the first two carbons of leucine

*Umbelopsis isabellina* growth on glucose media with/without ^13^C-formate demonstrates that formate as a supplemental carbon and energy source significantly (P < 0.05) improved the fungal biomass accumulation (Fig. 3a). At the end of the culture (48 h), the cultivations with and without formate reached 4.3 and 3.8 g L^−1^ biomass, respectively. The data also demonstrate that the formate and glucose were simultaneously consumed without glucose catabolite repression. Formate also influences intracellular NADH concentrations (Fig. 3b). NADH in the culture with formate peaked at 352 pmol mg^−1^ dry biomass at 24 h (in the middle-log phase growth). In contrast, the control culture without formate had a much lower NADH at the same growth phase. When glucose and formate were depleted at the end of fermentations, NADH contents in both cultures were decreased to ~110 pmol mg^−1^ dry biomass. This phenomenon confirms the role of formate as an auxiliary energy source to alleviate glucose catabolic burdens for NADH production and save glycolysis metabolites for biosynthesis [26]. Since glucose shows no catabolite repression on formate metabolism, the use of formate to relieve energy burden could be a preferable technique to improve performance of microbial cultivation [33].

During the culture on the glucose medium with ^13^C-formate, fungal proteinogenic amino acids demonstrate continuous ^13^C enrichment, especially for serine and methionine (Fig. 3c). Fractions of labeled serine and methionine reached to 20 and 30%, respectively, by the end of the culture. This result well aligns with the THF-mediated formate assimilation pathway that is closely related with the serine cycle and methionine metabolism [1]. Both of them facilitate the transmethylation reactions [34]. In addition, the experimental data show that percentages of *m* + 1 incorporated into aspartate and methionine (oxaloacetate-derived amino acids) were increased during the cultivation process (Fig. 3c). Aspartate labeling enrichment indicates that ^13^CO_2_ released from formate oxidation was re-incorporated into the TCA cycle via the anaplerotic reactions [35]. A BLAST was then conducted to search for the related enzymes in formate pathways of *U. isabellina* (Table 2). Key enzymes were blasted using a stand-alone database built from genomic sequence of *U. isabellina* and the tBLASTn search engine. The BLAST results confirmed enzymes involving in formate oxidation and assimilation (all e-values <10^−40^), demonstrating its native formate utilization capability.

**Table 2:** Key enzymes relevant to formate pathways identified by blasting the *U. isabellina* genome

Name	Reaction
NAD-dependent formate dehydrogenase (FDH, EC 1.2.1.1)	Formate + NAD^+^ ↔ CO_2_ + NADH + H^+^
Formate tetrahydrofolate ligase (EC6.3.4.3)	ATP + formate + tetrahydrofolate ↔ ADP + phosphate + 10-formyltetrahydrofolate
Methenyltetrahydrofolate cyclohydrolase/dehydrogenase (EC3.5.4.9 and 1.5.1.5/15)	5,10-Methenyltetrahydrofolate + H_2_O ↔ 10-formyltetrahydrofolate
Glycine dehydrogenase (EC1.4.1.10)	Glycine + H_2_O + NAD + ↔ glyoxylate + NH_3_ + NADH + H^+^
Aminomethyltransferase (EC2.1.2.10)	Glycine + tetrahydrofolate + NAD^+^ ↔ 5,10-methylene-tetrahydrofolate + ammonium + CO_2_ + NADH
Dihydrolipoyl dehydrogenase (EC1.8.1.4)	Protein N6-(dihydrolipoyl)lysine + NAD^+^ ↔ protein N6-(lipoyl)lysine + NADH + H^+^
Serine hydroxymethyltransferase (EC2.1.2.1)	5,10-Methylenetetrahydrofolate + glycine + H_2_O ↔ tetrahydrofolate + l-serine
Serine deaminase (EC4.3.1.17)	l-serine ↔ pyruvate + NH_3_

### Effects of formate on lipid synthesis

**Fig. 4:**
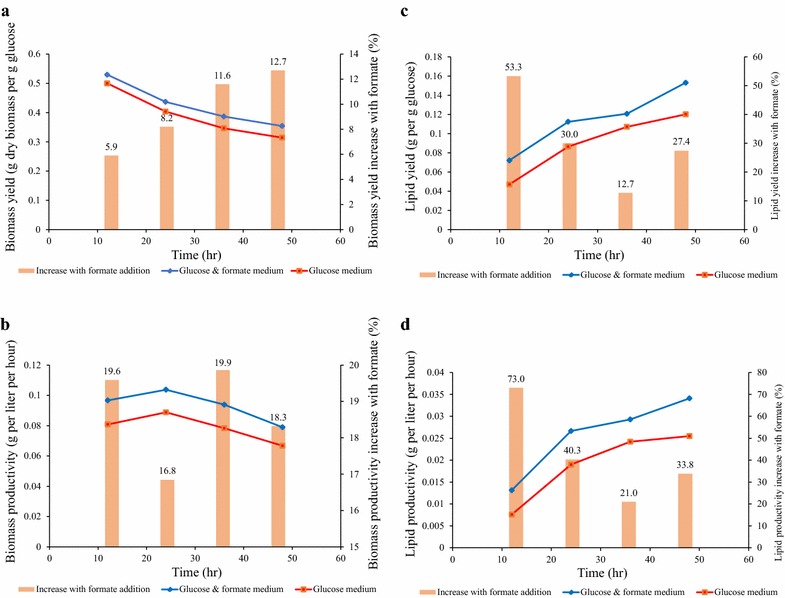
Enhancement of biomass and lipid accumulation by formate supplement. Data are average of two replicates with standard error. **a** Biomass yield; **b** biomass productivity; **c** lipid yield; **d** lipid productivity

Lipid accumulation during the fermentation was also influenced by formate (Fig. 4). The lipid contents in the fungal biomass at the end of cultivation reached 43 and 38 g lipid/100 g dry fungal biomass for the media with and without formate, respectively. Compared to the control culture (glucose medium only), the culture on the glucose plus formate medium significantly (P < 0.05) increased biomass yield (up to 13%), biomass productivity (up to 20%), lipid yield (up to 30%), and lipid productivity (up to 70%) (Fig. 4). As discussed in the previous section, formate co-metabolism alleviates the glucose dissimilation burden, and subsequently allows more glucose carbon to flow into lipid.

**Fig. 5:**
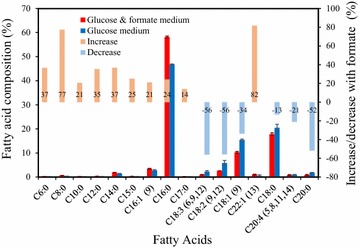
Fatty acid composition profile shift with formate. Data are average of two replicates with standard error

Interestingly, adding formate also changed the fatty acids profile (Fig. 5). The fatty acid biosynthesis includes recurring reactions that require a large amount of reducing factors and ATP [36]. The energy from formate oxidation may re-program fatty acid profiles. Short- and medium-chain fatty acids (C6:0 to C16:0) were all upregulated, while most of long-chain fatty acids (C17:0 to C22:1) were downregulated with the addition of formate (Fig. 5). Medium-chain fatty acids are widely used in detergents and lubricants due to their lathering and low-viscosity properties [37]. However, yeast species usually did not produce short- and medium-chain fatty acids except for engineered strains or cultures growing in synthetic media with high C/N ratios and low culture temperature [37]. Therefore, using formate to control fatty acid profile can be applicable in industrial fermentation processes.

### Flux balance analysis (FBA) of fungal growth on glucose and formate

**Fig. 6:**
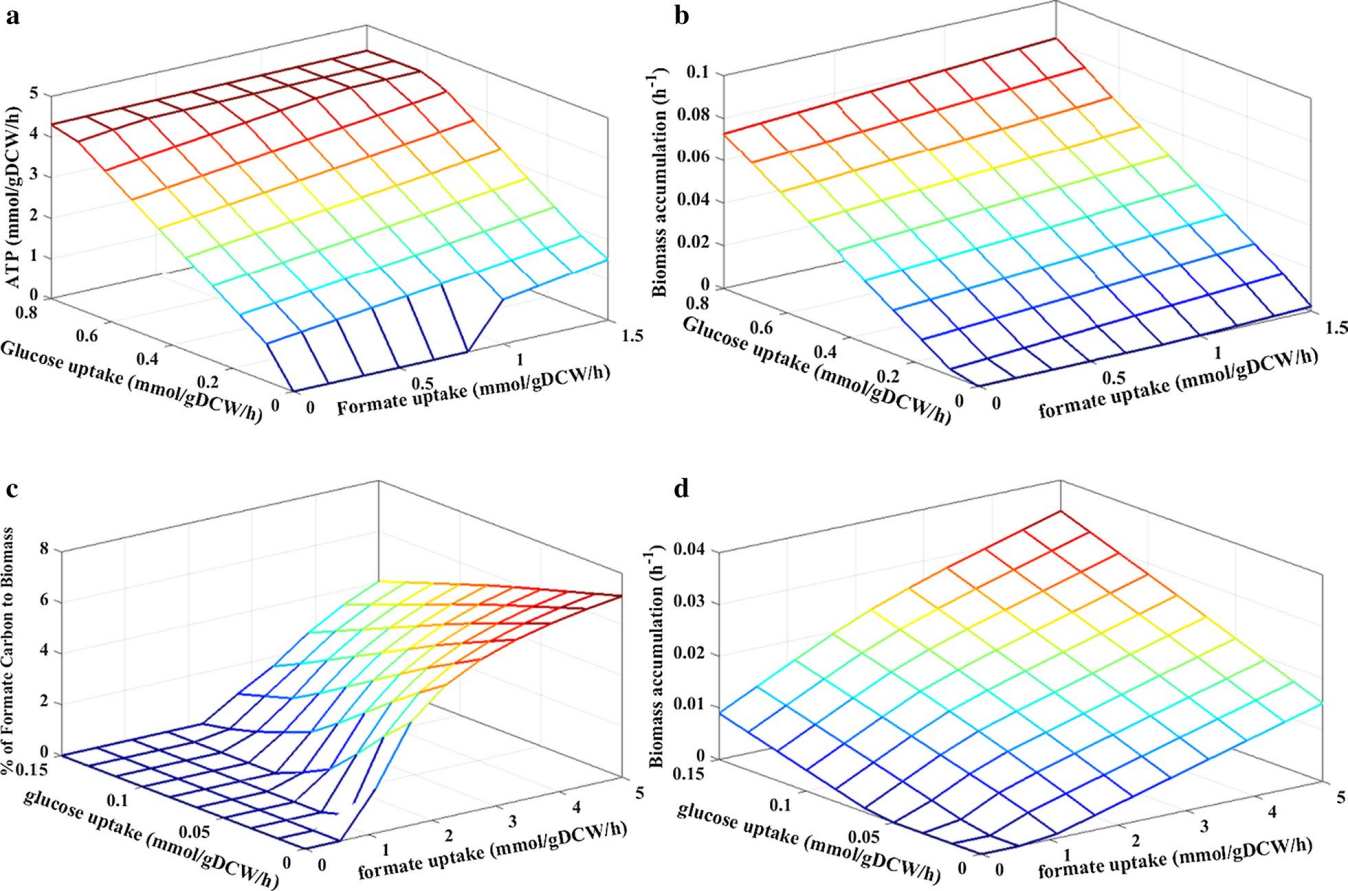
Results of FBA analysis. Oxygen flux is limited to 2 mmol g^−1^ DCW h^−1^ and maintenance flux is 1 mmol ATP g^−1^ DCW h^−1^. **a** Effects of formate and glucose utilization on ATP production. **b** Effects of formate and glucose uptake rates on biomass growth. **c** Percentage of formate carbon to biomass. **d** Effects of formate and glucose uptake rates on biomass growth at low glucose utilization rates

Glucose/formate uptake rates and biomass accumulation from the experiments were used to carry out FBA (Table 3). The FBA results verify the positive effects of formate on fungal biomass accumulation and ATP generations (Fig. 6). First, formate oxidation significantly contributes to ATP generation through respirations (formate → NADH → ATP), particularly at lower glucose uptake rate (Fig. 6a). ATPs were released from mitochondria when cell metabolism aerobically consumes both glucose and formate (Fig. 6a). Since this process requires a large amount of dissolved oxygen, high aeration is needed to maintain the formate oxidation of ATP generation. Second, the amount of formate carbon used for the biomass accumulation (through folate metabolism) depends on both glucose and formate uptake rates. Lower glucose and higher formate uptake rates are beneficial for formate assimilation (Fig. 6b, c, d). For instance, at high glucose uptake rates of 0.2–0.8 mmol glucose g^−1^ dry cell weight (DCW) h^−1^, formate was mainly used as the energy source and contributed little to biomass production  (Fig. 6b). Once the glucose uptake rate dropped to 0.15 mmol glucose g^−1^ DCW h^−1^, increasing formate utilization from 0 mmol glucose g^−1^ DCW h^−1^ to 5 mmol glucose g^−1^ DCW h^−1^ promoted biomass production (Fig. 6d). It is apparent that formate becomes both energy and carbon sources under glucose-limiting conditions (Fig. 6c). Third, the FBA also demonstrates in silico growth with formate in the absence of glucose (Fig. 6d). Under a high formate uptake rate of 5 mmol formate g^−1^ DCW h^−1^, the C1 assimilation pathways that *U. isabellina* possesses led to incorporation of 8% formate carbon into fungal biomass, while the remaining formate was oxidized as the energy source. The simulation results theoretically conclude the potential of formate as a carbon and energy source to support fungal growth. Although genetically modified organisms have been developed to use C1 feedstock, these engineered platform shows poor C1 utilizations (consumes less than 0.5 g L^−1^ C1 substrate) and slow fermentation processes [18–20]. *U. isabellina* offers an alternative non-model platform, and future metabolic engineering efforts should focus on development of genetic tools to re-program its metabolisms to produce diverse products by co-utilization of formate with other organic substrates.

**Table 3:** Formate and glucose uptake rates and biomass accumulation

Fermentation medium	Glucose uptake (mmol g^−1^ DCW h^−1^)		Formate uptake (mmol g^−1^ DCW h^−1^)		Biomass accumulation (mmol g^−1^ DCW h^−1^)	
	Glucose & formate medium	Glucose medium	Glucose & formate medium	Glucose medium	Glucose & formate medium	Glucose medium
3-L fermenter	0.609	0.576	0.499	–	0.054	0.031
3-L fermenter	0.505	0.577	0.816	–	0.027	0.037
0.7-L flask	0.484	0.397	0.345	–	0.012	0.010
0.7-L flask	0.026	–	0.117	–	0.017	–

## Conclusions

The results conclude that formate and glucose could be simultaneously utilized without catabolite repression. Formate was identified as a carbon and energy source in the culture of *U. isabellina* based on ^13^C-fingerprinting analysis and kinetic study. THF-based C1 assimilation pathways were counted for incorporation of formate into biomass. Extra NADH-dependent energy generated from formate oxidation led to enhanced biomass accumulation and elevated fatty acid synthesis as well as a significant shift in carbon chain length of the fatty acid profile. The FBA results further delineate the benefit of formate co-metabolism for fungal biomass accumulation. This study clearly presents a new microbial chassis that CO_2_ utilization (via formate) and lignocellulosic sugar conversion can be synergistically coupled to produce biofuels and value-added chemicals, so that overall carbon utilization efficiency can be significantly enhanced. Future studies should focus on genetic tools to modify *U. isabellina* for efficient utilization of formate and improvement of fatty acid profile.

## Abbreviations

CO_2_: carbon dioxide
ATP: adenosine triphosphate
NAD(P)H: reduced nicotinamide adenine dinucleotide
NADH: nicotinamide adenine dinucleotide
FDH: formate dehydrogenase
Methylene-THF: methylenetetrahydrofolate
PDA: potato dextrose agar
PDB: potato dextrose broth
YE: yeast extract
HPLC: high-performance liquid chromatography
PBS: phosphate buffered saline
GC–MS: gas chromatography–mass spectrometry
TBDMS: *N*-*tert*-butyldimethylsilyl-*N*-methyltrifluoroacetamide
FBA: flux balance analysis
DCW: dry cell weight
